# Curcumin Inhibits the Growth and Metastasis of Melanoma via miR-222-3p/SOX10/Notch Axis

**DOI:** 10.1155/2022/3129781

**Published:** 2022-05-09

**Authors:** Youqun Tang, Yanming Cao

**Affiliations:** Department of Oncology, The Third Xiangya Hospital, Central South University, Changsha, 410013 Hunan, China

## Abstract

**Background:**

The aim of this study was to investigate the effect of curcumin on melanoma and its mechanism.

**Methods:**

Curcumin (0, 0.125, 0.25, or 0.5 mg/ml) was utilized to treat A375 and HT144 cell lines. The MTT analysis was used to confirm the proliferation ability. Wound healing and transwell analysis showed the migration and invasion ability. Immunofluorescence assay was used to demonstrate the effect of curcumin on SOX10 expression. Multiple bioinformatic analysis to confirm the SOX10 associated miRNA. The correlation of miR-222-3p and SOX10 was detected by Luciferase reporter assays. qRT-PCR showed the miR-222-3p level. Western blot analyzed the expression of SOX10, Notch1, and HES1 in melanoma cell treated with or without miR-222-3p inhibitor.

**Results:**

Curcumin could inhibit the proliferation, migration, and invasion of melanoma cells. Furthermore, curcumin repress the expression of SOX10, Notch1, and HES-1, and increase the expression of miR-222-3p. And the miR-222-3p could directly target to SOX10 mRNA to inhibit its expression. In addition, inhibition of miR-222-3p expression reversed the inhibitory effect of curcumin the growth of melanoma cells.

**Conclusion:**

Curcumin enhances the miR-222-3p level to reduce SOX10 expression, and ultimately inactivates the Notch pathway in repressing melanoma proliferation, migration, and invasion.

## 1. Introduction

Melanoma is one of the most lethal cancers among all skin cancer with a markedly low survival rate, resulting in a serious social burden. To make things worse, there is still no life-saving treatment for patients with advanced melanoma. The five-year survival rate in the early stages of the disease exceeds 95%, but in the later stages, survival rarely exceeds a year [1, 2]. Although there are currently six new drugs approved for the treatment of advanced melanoma (https://www.drugs.com/mtm/trametinib.html; https://www.accessdata.fda.gov/drugsatfdadocs/label/2014/125514lbl.pdf; https://www.ono.co.jp/eng/news/pdf/smcn140704.pdf), they fail to improve patient survival and are expensive. Therefore, it is necessary to find novel drugs for the treatment of this disease.

Curcumin, a polyphenol from curcuma longa (turmeric), was an important spice, food coloring, and traditional herbal medicine in human history. Due to its antiinflammatory and antioxidant properties, curcumin has been also reported to have significant health benefits, including improved brain function and anticancer/anti-therosclerotic properties [3]. A previous study has been indicated that curcumin could inhibit breast cancer cells proliferation and invasion via repressing the NF-*κ*B inducing genes, including cyclinD and MMP-1 [4]. It has been suggested that curcumin could influence the G0/G1 phase arrest by MTA1- (metastasis-associated protein 1-) induced inactivation of Wnt/*β*-catenin pathway in the lung cancer cell [5]. In gastric cancer, curcumin could induce MMP proteins dissipation and cytochrome C into the cytosol in SGC-7901 cell lines, resulting in the downregulation of migration ability and apoptosis escaping [6]. In colon cancer HCT116 and HT29 cells, curcumin could inhibit the level and activity of hexokinase II (HKII) by a concentration-dependent manner, inducing cell apoptosis [7]. There is also an increasing number of studies showing that curcumin can also induce apoptosis and inhibit proliferation of melanoma cancer cells [8]. Therefore, this study conducted an in-depth study on the mechanism of curcumin on melanoma cells.

Sex determining region Y-BOX 10 (SOX10) is a transcription factor, which involved in the occurrence and development of various cancers [9]. It has been indicated that SOX10 impedes the immunogenicity of melanoma cells through the IRF4-IRF1 axis [10]. In our previous study, we found that SOX10 mRNA and protein expression was obviously increased in melanoma tissue samples compared to normal tissue samples, which could significantly promote the melanoma cell proliferation via activating Notch pathway [11].

MicroRNAs (miRNAs) are noncoding RNAs that participate in a variety of cellular processes and can inhibit or promote tumorigenesis. At present, some miRNAs have been found to play a role in melanoma. For example, MiR-205 is cancer suppressor miRNA that is downregulated in melanoma [12], which is related to cell survival and invasion [13]. MiR-221/222 is also an oncogenic miRNA cluster that is often overexpressed in melanoma and enhances the proliferation of cancer cells [14].

Previous study was founding the tumor-suppressing effects of miR-222-3p have shown that miR-222-3p inhibited the level of PDCD10 to decrease the tumor metastasis via regulating Wnt/*β*-catenin signaling pathway in ovarian cancer [15]. Fu et al. found that the miRNA-222-3p/GNAI2/AKT axis inhibited epithelial ovarian cancer cell proliferation and migration [16]. However, it is unknown whether curcumin affects the expression of miR-222-3p and SOX10, and whether there is interaction between miR-222-3p and SOX10 in melanoma. Therefore, in this study, melanoma cells were used to investigate the mechanism of curcumin on melanoma in vitro, as well as the role of miR-222-3p and SOX10 in it, aiming to provide new therapeutic options for the clinical treatment of melanoma.

## 2. Methods

### 2.1. Bioinformatic Analysis

LinkedOmics (http://www.linkedomics.org/login.php), a publicly available database, includes 32 cancer types datasets based on TCGA database and 10 cancer types datasets based on CPTAC database [10]. This database was used to analysis the miRNAs correlated with SOX10 mRNA level. TargetScan (http://www.targetscan.org) was used to predict biological targets of miRNAs associated with SOX10 by searching for the presence of conserved sites that match the seed region of each miRNA [11]. The study was approved by the ethics committees of The Third Xiangya Hospital, Central South University.

### 2.2. Cell Culture and Treatment

A375 and HT144 cells were purchased from National Collection of Authenticated Cell Cultures. A375 and HT144 cell cultures were performed referring to our previous study [10]. The cells were cultured in DMEM medium containing 10% fetal bovine serum (FBS) and 1% penicillin-streptomycin in a 5% CO2 incubator at 37 °C.

These cells were treated with different curcumin (S1848, Selleck) concentrations (0, 0.125, 0.25, or 0.5 mg/ml) for 48 hours at 37 degree centigrade. miRNA-222-3p mimics or inhibitors and SOX10 overexpression vectors were purchased from RiboBio (Guangzhou, China). The cells were seeded in 6-well plates and then transfected with 50 nM of miRNA mimic/NC, inhibitor/NC, and 500 ng recombinant plasmid, respectively, using Lipofectamine 2000 (Invitrogen, Carlsbad, CA, USA). After 6 hours of transfection, the culture medium was replaced with fresh medium containing 10% FBS. All transfections were performed for 48 h, prior to harvesting for the following assays.

### 2.3. MTT Analysis

MTT analyses were performed as previously described [10]. Cells were seeded into 96-well plates at a density of 6 × 10^3^/well. After adhering to the wall, curcumin was treated for 24, 48, and 72 h, respectively, Add 20 *μ*L 5 mg/mL MTT solution (Gefan Biotechnology Co., Ltd., Shanghai, China), and incubate in incubator for 4 hours. Discard the medium, and add 150 *μ*L DMSO to dissolve the crystal. The absorbance at OD450 was measured using an enzyme marker (SpectraMax M5, Molecular Devices, CA, USA).

### 2.4. Wound Healing Analysis

The cells were inoculated in 6-well plate at the density of 5 × 10^5^/well and adhered to the wall for 24 hours. The cells were scratched with a pipette tip. At 0 h and 48 h, the wound was observed and photographed using a light microscope (Olympus), and the wound healing rate was calculated.

### 2.5. Transwell Assays

The A375 or HT144 cells were digested and resuspended in DMEM medium, then 2.5 × 10^5^ cells were counted and inoculated in the upper chamber precoated with matrixgel in the 24-well plates, and 750 *μ*l of DMEM containing 10% FBS was added to the lower chamber and cultured at 37 °C. After 24 h, 4% paraformaldehyde was used to fix the migrated cells for 30 min and dyed with 5% crystal violet for 15 min. A cotton swab was then used to remove the un-migrated cells in the upper chamber. Observe the cells under a microscope and take pictures.

### 2.6. Luciferase Reporter Assays

SOX10-3′UTR with or without mutation was subcloned into pGL3 promoter plasmid. 30,000 cells were seed into 24-well culture plates in phenol red-free medium. After transfection with miRNA-NC or miR-222-3p mimics for 48 h, the luciferase activity was determined by the Dual Luciferase Assay Kit (Promega, Madison, WI, USA) according to the manufacturer's instructions.

### 2.7. Quantitative Real-Time PCR

Quantitative real-time PCR assays were performed as previously described [10]. Using TRIZOL regent (Thermo Fisher Scientific) to extract total RNA of cells, the extracted mRNA was reverse transcribed into cDNA using the cDNA Reverse Transcription Kit (Thermo Fisher Scientific). SYBR Green PCR kit (TaKaRa, China) was used for qRT-PCR. The relative mRNA expression was calculated according to the 2−*ΔΔ*Ct method. Take GAPDH as the internal reference. Primer sequences used in this qRT-PCR are as follows: miR-222-3p: forward: 5-ACACTCCAGCTGGGAGCTACATCTGGCTACTG-3, reverse: 5-CTCAACTGGTGTCGTGGA-3; U6: forward: 5-CTCGCTTCGGCAGCACA-3, reverse: 5-AACGCTTCACGAATTTGCGT-3; SOX10: forward: 5-CCTCACAGATCGCCTACACC-3, reverse: 5-CATATAGGAGAAGGCCGAGTAGA-3; and GAPDH: forward: 5-GTCT CCTCTGACTTCAACAGCG-3, reverse: 5-ACCACCCTGTTGCTGTAGCCAA-3.

### 2.8. Western Blot

The total protein was isolated from cells and tissues using RIPA lysis buffer (Santa Cruz, Dallas, USA). Protein samples were run on sodium dodecyl sulfate polyacrylamide gel electrophoresis (SDS-PAGE) and transferred to polyvinylidene fluoride (PVDF) membranes (Thermo Fisher Scientific). After blocking the PVDF membrane in TBST with 5% skim milk, add primary antibody SOX10 (ab227684, Abcam), Notch1 (ab52627, Abcam), Hes1 (ab108937, Abcam), and GAPDH (ab8245, Abcam), and incubate overnight on a shaker at 4 °C. PVDF membrane was incubated with horseradish peroxidase-conjugated secondary antibody for 2 h at room temperature. After adding luminescent solution, the protein bands were visualized using the ECL system (Thermo Fisher Scientific), and the grayscale statistics was carried out by Image J software (Bio-Rad, USA). GAPDH is used as an internal reference.

### 2.9. Immunofluorescence

These A375 and HT144 cells were fixed with 4% paraformaldehyde preheated at 37 degree centigrade for 30 min at room temperature (RT). The cells were permeabilized by treating with 0.5% Triton X-100. Subsequently, cells were blocked in 5% normal goat serum (ZSGB-BIO) for 60 min at 37 degree centigrade and then incubated with SOX10 antibodies diluted 1 : 50 in PBST for overnight at 4 degree centigrade. Next day, the cells were washed with PBS and incubated with the secondary fluorescent antibody (diluted 1 : 500 with PBS) for 60 min and then stained with DAPI for 5 min. Finally, the cells were imaged using an inverted fluorescence microscope.

### 2.10. Statistical Analysis

All data analyses were performed using SPSS 24.0 software (SPSS, Inc., Chicago, IL, USA). The data were expressed as mean ± standard deviation (SD). One-way ANOVA was used to analyze the differences between multiple groups, and T test was used to analyze the differences between two groups. *P* < 0.05 was considered a significant difference.

## 3. Results

### 3.1. Curcumin Significantly Inhibit the Malignant Progression of Melanoma Cells

**Figure 1:**
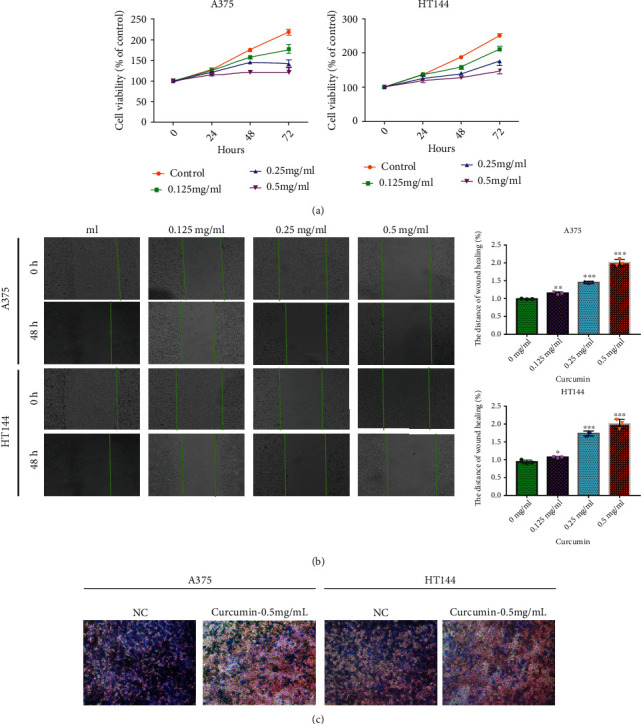
The anticancer effect of curcumin on melanoma cell. (a) The effect of different concentration of curcumin on A375 and HT144 cells via MTT analysis. (b) The migration ability of different concentration of curcumin on A375 and HT144 cells via wound healing analysis. (c) The invasion ability of different concentrations of curcumin on A375 and HT144 cells via transwell assays. ∗∗*P* < 0.01, ∗∗∗*P* < 0.001 vs. control group.

To confirm whether the pharmacologic effect of curcumin on the melanoma cells malignant progression, we utilized the 0.125 mg/ml, 0.25 mg/ml, and 0.5 mg/ml curcumin to test the cytotoxicity of curcumin in A375 and HT144 cell lines. We found that curcumin significantly decreased A375 and HT144 cell viability in a dose-dependent manner. Moreover, the 0.5 mg/ml curcumin has the strongest inhibitory effect on A375 and HT144 cell lines (Figure 1(a)). Subsequently, we found that the inhibition of curcumin on melanoma cell migration was also by a dose-dependent manner (Figure 1(b)), which 0.5 mg/ml curcumin had the best pharmacologic effects. The effect of 0.5 mg/ml curcumin on the invasion of melanoma cells was further determined by transwell. The results showed that 0.5 mg/ml curcumin could obviously reduce the invasion cell numbers compared to NC group (Figure 1(c)). These results indicated that curcumin had an important anticancer effect on melanoma cell.

### 3.2. SOX10 Can Interact with miRNA-222-3p in Melanoma Cells

**Figure 2:**
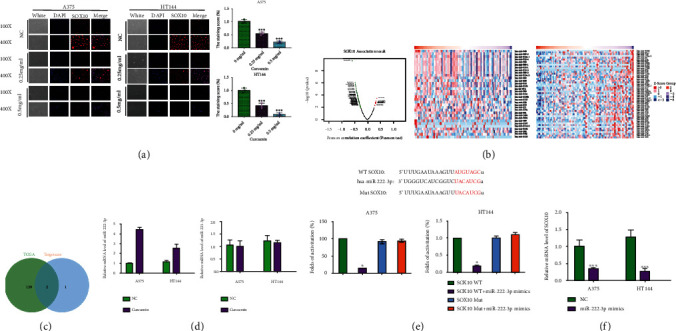
The effect of curcumin on SOX10 and miR-222-3p. (a) The effect of different concentrations of curcumin on SOX10 expression in A375 and HT144 cells. (b) Volcano plot and heat map of the miRNAs correlated by SOX10 in TCGA database. (c) Prediction potential targets of miR-222-3p and miR-221-3p assessed by TargetScan and TCGA. (d) The effect of curcumin on miR-222-3p and miR-221-3p level in A375 and HT144 cells via qRT-PCR analysis, ∗*P* < 0.05 vs. NC group. (e) Luciferase reporter to verify the interaction between SOX10 and miR-222-3p, ∗*P* < 0.05 vs. SOX10 WT group. (f) The expression level of SOX10 in cells after overexpressed of miR-222-3p was detected by qRT-PCR, ∗∗∗*P* < 0.001 vs. NC group.

In our previous study, we found that sex-determining region Y-related high mobility group-box 10 (SOX10) was an oncogenetic role in melanoma progression, especially in proliferation [10]. Therefore, we detected the SOX10 expression in curcumin groups and NC group by immunofluorescence analysis, which showed that the expression of SOX10 was significantly decreased in curcumin groups compared to NC group by a dose-dependent manner (Figure 2(a)). In order to further explore the molecular mechanism of curcumin regulating SOX10, we made a correlation analysis among SOX10 and miRNAs based on TCGA database Melanoma datasets. We found 141 miRNAs were significantly and negatively correlated with SOX10 mRNA level (Figure 2(b)). Furthermore, we extracted miRNAs datasets that targeted SOX10 based on TargetScan database (http://www.targetscan.org). Then, the miRNAs between TCGA and TargetScan were generated by using Venn diagram (Figure 2(c)), which showed that 2 miRNAs, miRNA-222-3p and miRNA-221-3p, were negatively and directly correlated with SOX10 in melanoma patients. Furthermore, qRT-PCR was used to verify the expression of miRNA-222-3p and miRNA-221-3p in melanoma cells and its interaction with SOX10. The results indicated that miRNA-222-3p was significantly increased after curcumin treatment, but miRNA-221-3p was not significantly influenced by curcumin (Figure 2(d)). To demonstrate the direct effect of miRNA-222-3p on SOX10, a luciferase assay was performed. The results showed that the overexpression of miRNA-222-3p could significantly inhibit the expression of SOX10, while SOX10 mutation binding sites was not affected by miRNA-222-3p mimics (Figure 2(e)). Moreover, the mRNA level of SOX10 was significantly decreased by miRNA-222-3p mimics (Figure 2(f)).

### 3.3. Inhibition of miRNA-222-3p Can Reverse the anti-Tumor Effect of Curcumin

**Figure 3:**
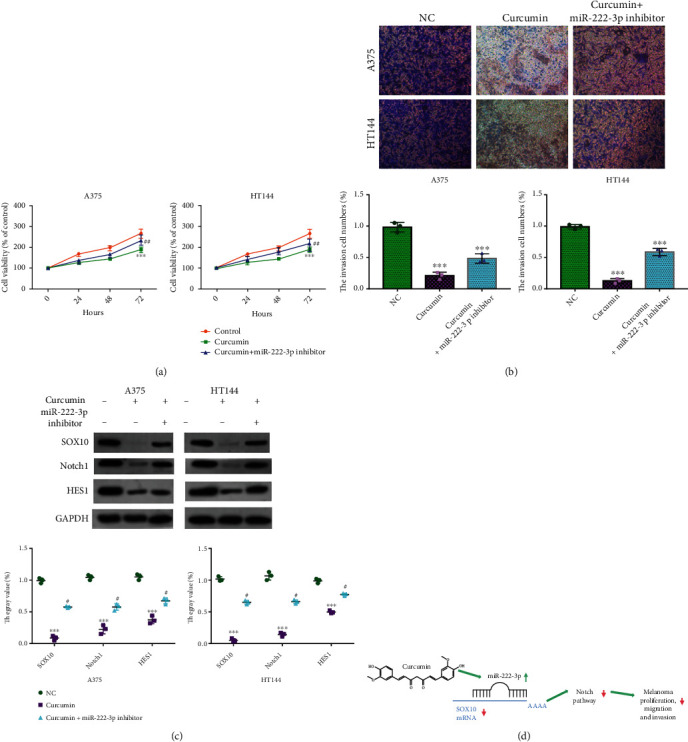
Curcumin enhance miR-222-3p to inhibit SOX10 via inactivating Notch pathway. (a) The proliferation of A375 and HT144 cells in each group was detected by MTT. ∗∗∗*P* < 0.001 vs. Control group, ##*P* < 0.01 vs. curcumin group. (b) The invasion ability of A375 and HT144 cell in each group was detected by transwell. (c) The expression of SOX10, Notch1, and and HES1 in A375 and HT144 cell groups was detected by Western blot. (d) A model of the molecular mechanism of the curcumin/miR-222-3p/SOX10/Notch signaling pathway.

Moreover, we further detected the role of miRNA-222-3p in the progression of melanoma. We treated A375 and HT144 cell lines with curcumin or curcumin plus miRNA-222-3p inhibitor (Figure 3(a)). The cell proliferation was significantly decreased by curcumin, which could be reversed by miRNA-222-3p inhibitor. The invasion ability of A375 and HT144 cell was also decreased by miRNA-222-3p but reversed by miRNA-222-3p inhibitor (Figure 3(b)). We further found curcumin could inhibit the expression of SOX10, Notch1, and Hes1, which could be reversed by miRNA-222-3p inhibitor (Figure 3(c)). Taken together, the curcumin enhances the miR-222-3p level to inhibit SOX10 expression, which inactivates the Notch pathway to impede the melanoma cell proliferation, migration, and invasion (Figure 3(d)).

## 4. Discussion

This study indicated the effect of curcumin on melanoma cell lines, including A375 and HT144 cells. We found that exposure to curcumin obviously repressed proliferation, migration, and invasion in melanoma cells. The proliferation, migration, and invasion ability were significantly decreased after treated with curcumin in a dose-dependent manner in melanoma cells. In the previous study, curcumin could enhance miR-98 level to inhibit LIN28A mRNA level, resulting in suppressing lung cancer cell growth and invasion [17]. Curcumin could also inhibit miR-7641 to enhance p16 expression, which led to the downregulated invasion and apoptosis escaping of bladder cancer cells [18]. Dou et al. found that curcumin inactivated Wnt/beta-catenin pathway to repress cell proliferation in colon cancer via enhancing miR-130a [19]. Our study indicated that curcumin serves a key role in the suppression of melanoma cell proliferation, migration, and invasion via enhancing miR-222-3p which targeted to SOX10. Furthermore, miR-222-3p inhibitor markedly reversed the effect of curcumin on melanoma cellular proliferation, migration, and invasion. Taken together, these results indicated that the miRNAs regulation was the significant way in anticancer molecular mechanisms for curcumin.

Melanoma is the most fatal form of malignant skin cancer with high invasiveness and arises from melanocytes [20]. At the molecular and cellular levels, curcumin inactivates EMT cascade and affects many targets involved in melanoma initiation and progression (e.g., BCl-2, MAPKS, P21, and some microRNAs) [21], which emerge with great potential for clinical applications. In this study, we found that the miR-222-3p/SOX10/Notch pathway was considered one of the most important mechanisms of the anticancer effect of curcumin. miR-222-3p played an anticancer role in ovarian cancer [22], but served as a oncogene in thyroid carcinoma [23], lung cancer [24], and osteosarcoma [25]. The paradoxical role may lie in the differential transcription levels of key downstream genes targeted by miR-222-3p in different cancer types, especially in melanoma. SOX10, an essential transcriptional factor, belongs to the SOX family of transcription factors, which involved in the occurrence, development, and progression of multiple cancer types [26]. We identified that curcumin had a repress effect on SOX10 expression to impede the proliferation, migration, and invasion ability via a dose-dependent manner in melanoma cells. These results are consistent with previous studies, indicating that curcumin have an antitumous effect.

The Notch signaling pathway, an oncogene in melanomagenesis, was involved in cell fate decisions, tissue patterning, and morphogenesis during development, which was an evolutionarily conserved, intercellular signaling cascade [27]. Notch1 deficiency sensitizes melanoma cells to stress-induced cell death and repress proliferation ability in part by inhibiting CCND1, resulting in decreasing in melanoma growth [28]. Moreover, Notch pathway could interact with ERBB pathway [29], Nodal pathway [30], or canonical Wnt signaling pathway [31] to promote melanoma progression. Furthermore, He et al. found that curcumin could target Notch pathway to decelerate the development and progression of cervical cancer [32]. Yang and his colleagues suggested that curcumin could impede prostate cancer cells survival and metastasis via inactivating Notch-1 signaling pathway [33]. Curcumin could also mediate the death of esophageal cancer cells by modulating Notch signaling [34]. In hepatic cancer, curcumin promotes cell apoptosis and proliferation arrest through Notch pathway [35]. Similar results were observed in the study about the effect of curcumin on osteosarcoma cells [36]. The present study found curcumin inactivates Notch signaling pathway by regulating miR-222-3p/SOX10 axis in melanoma cell. Thus, our study shows that the inhibition of Notch signaling by curcumin may be highly conserved in multiple tumor types and further explains how curcumin regulates Notch signaling at the molecular level.

## 5. Conclusion

In summary, these results indicated that curcumin was a key role in impeding the proliferation, migration, and invasion ability of melanoma cells. Curcumin enhances the miR-222-3p level to reduce SOX10 expression and ultimately inactivates the Notch pathway in melanoma. According to the above, curcumin represents a potential therapeutic agent for the treatment of melanoma.

## Data Availability

The data used to support the findings of this study are available from the corresponding author upon request.
